# Differences in gait parameters when crossing real versus projected everyday life obstacles in healthy children and adolescents

**DOI:** 10.1038/s41598-023-34276-8

**Published:** 2023-05-15

**Authors:** Sabrina Gröble, Hubertus J. A. van Hedel, Jeffrey W. Keller, Corinne Ammann-Reiffer

**Affiliations:** 1 Swiss Children’s Rehab, University Children’s Hospital Zurich, Mühlebergstrasse 104, 8910 Affoltern am Albis, Switzerland; 2 Children’s Research Center, University Children’s Hospital Zurich, University of Zurich, Steinwiesstrasse 75, 8032 Zurich, Switzerland; 3 Department of Health Sciences and Technology, ETH Zurich, Leopold-Ruzicka-Weg 4, 8093 Zurich, Switzerland; 4 Department of Health Professions, Applied Research & Development in Nursing, Bern University of Applied Sciences, Murtenstrasse 10, 3008 Bern, Switzerland; 5 Federal Office of Public Health FOPH, Schwarzenburgstrasse 157, 3003 Bern, Switzerland

**Keywords:** Therapeutics, Paediatric research

## Abstract

Practicing complex everyday life walking activities is challenging in paediatric neurorehabilitation, although it would prepare patients more comprehensively for the requirements of daily life. Floor projections allow simulation and training of such situations in therapy. Twenty healthy youths aged 6–18 years stepped over a tree trunk and balanced over kerbstones in a real and projected condition. Spatiotemporal and kinematic parameters of the two conditions were compared by equivalence analysis, using the medians of the differences between the two conditions with their bootstrapped 95% confidence intervals. Velocity, step and stride length, step width, and single support time were generally equivalent between the two conditions. Knee and hip joint angles and toe clearance decreased substantially during the execution phase of the projected tree trunk condition. The largest differences were found at the end of the execution phase in both tasks for the ankle joints. As spatiotemporal parameters were equivalent between the conditions, floor projections seem suitable to train precise foot placement. However, differences in knee and hip joint kinematics and toe clearance revealed that floor projections are not applicable for obstacles with a vertical extension. Therefore, exercises aiming at knee and hip flexion improvement should favourably be trained with real objects.

## Introduction

In paediatric neurorehabilitation, children and adolescents with various acquired or congenital neurological disorders, such as cerebral palsy, traumatic brain injuries, stroke, or spinal cord injuries, are treated. As these central nervous system pathologies can impair the sensorimotor system, walking ability is often limited in these children^1–3^. Therefore, improving or restoring walking ability is one of the most important therapy goals for the children and their families as it allows them to participate in activities of daily life^4,5^. In neurorehabilitation programs, conventional physical therapy and robot-assisted gait training can complement each other to provide the best possible therapy for each child^5,6^. Exergames, which are ‘video games that require the player to physically move in order to play’^7^, are regularly used, for example, during robot-assisted training of the upper and lower limbs^8–11^. More recent developments include augmented (AR) and virtual reality (VR) systems^12,13^. In AR, virtual objects are added to the real world in real-time during the user’s experience^14^. VR replicates an environment that simulates a physical presence in the virtual world, thus creating a fully immersive experience^14^. While such systems are not yet used in standard therapies, their potentially beneficial impact is clear. For example, it is easy to adapt the degree of challenge to the patient’s needs^15^ and keep children more motivated during repetitive tasks^8^. Ideally, these systems should also automatically collect data during the rehabilitation process without requiring any additional assessment. These aspects would allow the continuous adaptation of therapies to the abilities of the individual patient, thus enhancing the outcome^16^.

Projecting tasks on the floor is another recent approach that has found its application in clinical studies. Up to now, floor projections were often two-dimensional and mainly used to modify footprint parameters^17^, regulate gait patterns^18,19^, or improve balance and muscle strength to prevent falls^20,21^. All these examples are restricted to the training of body functions as defined by the International Classification of Functioning, Disability and Health (ICF) by the World Health Organization^22^. However, it has become increasingly important to define therapy goals at the ICF activity level^23^ and transfer functional improvements achieved during the rehabilitation program to daily life activities^16^.

Related to walking, it is still challenging to train more complex everyday life walking activities, such as crossing a street or dodging people in a large crowd, in a location-bound rehabilitation setting. Nevertheless, training such activities would allow patients to prepare for their daily life requirements more comprehensively. A transfer may be enhanced by practising everyday life walking scenarios with AR or VR, which still needs some technical advances to its clinical implementation. However, first steps towards such an approach are being taken, for example by Binaee and Diaz^24^, who generated a perception of a three-dimensional obstacle from a two-dimensional floor projection whose perspective was adapted according to the subject’s motion-tracked head position. The three-dimensional effect was thereby further improved using stereoscopic goggles. In that study, the authors investigated differences in gait behaviour of young, healthy adults while stepping over these three-dimensional AR obstacles of different heights and similar physical obstacles. They found a slower approach speed, an increased toe clearance above the obstacle, and a larger distance between the lead toe and the obstacle for AR than for real obstacles. In another study, Huang et al.^25^ used virtual obstacles projected on a cylindrical screen in front of a treadmill to investigate different spatiotemporal gait adjustments in adults with Type 2 Diabetes Mellitus and healthy controls when walking on a treadmill. Their results showed that the success rate in crossing a virtual obstacle was higher for the leading limb (LL) than for the trailing limb (TL) in both groups. Further studies used VR provided through head-mounted displays or immersive CAVE-like environments to explore gait adaptations or obstacle avoidance in a highly standardized way^26–28^.

However, the existing studies exclusively focused on adults. Further, most of the above mentioned technologies are not yet applicable or affordable for the clinical setting. We therefore aimed to explore a low-cost application that therapists could use in everyday clinical practice without much additional effort. We selected the use of floor projections provided by a standard projector mounted to the ceiling as a low-cost application to train everyday life walking activities in a rehabilitation setting. To evaluate the suitability of this easy-to-use approach, we brought two obstacles from everyday life into the gait laboratory and presented them in real and as three-dimensional floor projections. We thereby wanted to examine to which extent different spatiotemporal and kinematic gait parameters of the lower limbs of healthy children and adolescents between 6 and 18 years of age are comparable while crossing a tree trunk and balancing on a kerbstone in a real and projected condition.

Based on our professional experience in working with children and our own test experience, we hypothesized that gait velocity and step length would be similar for real and projected obstacles in both scenarios. Additionally, we assumed that step width and single support time would be comparable in the kerbstone scenario. Further, we expected similar ankle joint angles for the two conditions in the kerbstones scenario. At the same time, we assumed decreased maximal toe clearance and ankle, knee, and hip joint angles for the projected tree trunk scenario (as the three-dimensional appearance of the trunk got lost at the point where participants crossed it).

## Materials and methods

### Study design and setting

The measurements of this cross-sectional study were performed in the gait laboratory of the Swiss Children’s Rehab of the University Children’s Hospital Zurich in Affoltern am Albis, Switzerland. The single appointment lasted 60–90 min. According to the clarification of responsibility (Req-2018-00657) the ethics committee of the Canton of Zurich, Switzerland, waived the need for ethical approval for the study. All methods were performed in accordance with the relevant guidelines and regulations.

### Participants

Healthy children and adolescents aged 6–18 years were recruited through flyers and announcements in the in-house research newsletter. The participants and their parents were informed verbally and in writing and provided informed consent and assent. Excluded were children and adolescents with a neurological diagnosis, musculoskeletal deficits that were likely to affect balance or mobility, vestibular disorders, restriction of visual acuity, and injury or surgery at the lower extremities within the last year. The participants were kept unaware of the specific study aims to prevent them from consciously influencing the measurements.

### Experimental setup

#### Design of the scenarios

**Figure 1:**
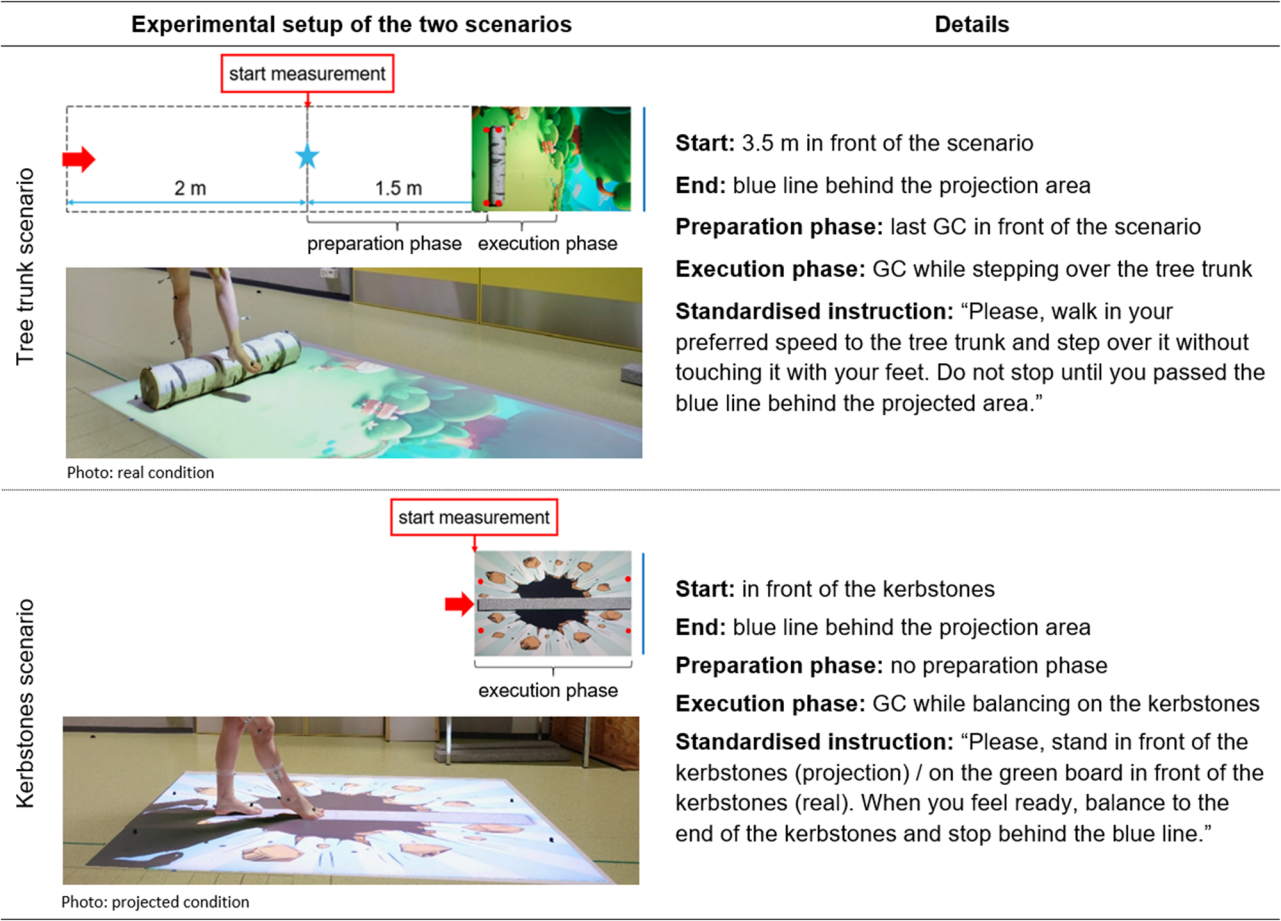
Scenarios (schematic top view, photos lateral view), experimental setup, and standardised instructions. The participants had to (1) step over a tree trunk^29^ and (2) balance on a line of kerbstones^30^. Both obstacles were presented in real and as a projection. Explanation of the signs used: red arrows = start of the trials, red dots = marker positions, blue star = point where the two conditions looked as similar as possible. *Abbreviations GC* gait cycle.

Two different objects were presented to the participants as a real obstacle and as a floor projection (Fig. 1): a tree trunk (height: 0.15 m, length: 0.85 m, width: 0.18 m) and a line of kerbstones (height: 0.07 m, length: 0.2 m, width: 0.13 m). For the floor projection, a photo of both obstacles was taken and edited in Adobe Photoshop CC 2018© (Adobe, San Jose, CA, USA) to create the best possible three-dimensional picture. The length and width dimensions of the obstacles were the same in the real and the projected condition. To improve the three-dimensional effect and increase the motivation of the participants, both conditions were enriched with cartoon-style backgrounds (tree trunk^29^, line of kerbstones^30^, Fig. 1). These backgrounds and the obstacles in the projected condition were projected onto the floor with a standard projector (EPSON EH-TW3200, SEIKO Epson CORPORATION, Japan) mounted to the ceiling. A customized mirror was attached at a 45°-angle in front of the projector’s lens. A white matt foil, which was stuck to the floor of the gait laboratory, prevented light reflexions, and assured high-quality projections.

With the available technical equipment, it was not possible to change the obstacles’ appearance whilst the participant approached. Therefore, the picture for the projection of the tree trunk was edited that it looked as similar as possible to the real trunk when standing at a distance of 1.50 m in front of the object (blue star Fig. 1). This distance allowed capturing at least one gait cycle in front of the scenario with the participant seeing both obstacles, the real and the projected one, in a similar representation.

### Measurement procedure

Eighteen infrared reflective markers (diameter: 16 mm) were placed on specific anatomical landmarks of the participants’ lower limbs and pelvis according to the Helen Hayes marker set^31^. Four additional markers (red dots Fig. 1) tagged the position of the obstacle for later calculations. The participants had to step over the tree trunk and balance on the kerbstones (Fig. 1), both presented as real obstacles and as a projection, receiving standardised instructions beforehand. We expected that the order in which the obstacles and conditions were presented might affect the participants’ trial execution, so we randomized the order using sealed opaque envelopes. When the scenarios and conditions were changed, the participants remained in the room.

The trials of the tree trunk scenario started 3.50 m in front of the obstacle (red arrow). The first two metres served for acceleration. We chose the 2 m for acceleration based on the 10-m gait test, which specifies an acceleration phase of 2 m to assess steady-state walking speed in various patient groups^32^. At a distance of 1.50 m (blue star) in front of the tree trunk, where the scenarios looked as similar as possible in the two conditions, data capturing started with the preparation phase and ended with the execution phase. The trials of the kerbstones scenario started directly in front of the kerbstones (red arrow). Thus, only an execution phase with two gait cycles was measured. For each scenario and condition, eight valid trials starting with the same leg were recorded while the participants walked barefoot at their self-selected speed. The participants were not instructed to begin with a specific leg as their walking should not be influenced. Therefore, the measurements were continued until eight valid trials were captured for one of the two legs. A trial was valid unless the tree trunk was touched, the participants lost balance on the kerb stone, ran or jumped, or there were technical problems (e.g., missing markers in the motion capture software, late start, or early stop of the recordings). The first six valid trials were used for data analysis, with two additional trials available in case of any technical problems.

### Outcome parameters

Spatiotemporal and kinematic outcome parameters (Table 1) were recorded utilizing 12 Vicon Vero 2.2 high-speed cameras (Vicon Motion Systems, Oxford, UK) at a frequency of 100 Hz and processed with Vicon Nexus Motion Capture Software (Vicon Motion Systems, Oxford, UK). Marker trajectories were smoothed using a Woltering filter with a mean squared error value of 20. Simultaneously, two VICON VUE high-speed cameras (1080p frame-synchronized colour video overlay, Vicon Motion Systems, Oxford, UK) were positioned in the sagittal and frontal plane to clarify potential uncertainties or inconsistencies in the outcome parameters.

**Table 1:** Overview of the assessed spatiotemporal and kinematic gait parameters.

Phase	Parameter		Tree trunk	Kerbstones
Preparation	Spatiotemporal parameters	Velocity (m/s)	✓	
		Step length (m)	✓	
Execution	Spatiotemporal parameters	Velocity (m/s)		✓
		Step length (m)	✓	✓
		Step width (m)		✓
		Stride length (m)	✓	
		Single support time (s)		✓
		Maximal toe clearance (m)	✓	
	Kinematic parameters	Minimal ankle joint angles (°)	✓	✓
		Maximal ankle joint angles (°)	✓	✓
		Maximal knee joint angles (°)	✓	
		Maximal hip joint angles (°)	✓	

We focused on the marked parameters.

The trials were divided into a preparation phase and an execution phase for the tree trunk scenario, and an execution phase with two gait cycles for the kerbstones scenario (Fig. 1). Relevant spatiotemporal and kinematic outcome parameters were selected based on the literature (Table 1)^33,34^. Where relevant, parameters were assessed for the LL, which crossed the obstacles first, and the TL separately. Detailed information on how the parameters were calculated from the marker data can be obtained from the corresponding author.

### Statistical analysis

All outcome parameters were calculated with MATLAB (MathWorks, Natick, US), and statistical analyses were performed using the package “boot”^35^ in R version 3.5.1 (R foundation, Wien, Austria) and SPSS version 25 (IBM SPSS Statistics, Armonk, NY, USA). Descriptive statistics are presented for relevant characteristics of the study participants and the outcome parameters. To gain insight into how the participants the tasks, the trajectories of both ankle markers of all participants were plotted and visually analysed.

For each parameter, the difference between the means of six valid trials of the projected and the real condition was calculated for each participant. We calculated the median value of the differences with the bootstrapped 95% confidence interval (95% CI; bias-corrected and accelerated, 2’000 samples) for each outcome measure.

To evaluate the comparability of the two conditions, we performed equivalence analyses. Equivalence analyses are generally used to investigate if a novel therapy is similarly effective as the current standard treatment^36^. In our study, the equivalence margins were set for each gait parameter individually, according to reference values from the literature. The standard deviation (± 1 SD) of test–retest reliability studies evaluating level ground walking in healthy children and adolescents^37,38^, or if not available, young adults^37^, respectively, constituted the margins. These margins served as boundaries that the 95% CI of the difference of the medians between the conditions should not cross. Therefore, if the 95% CI lay within the boundaries of variability that 66% of youths or young adults show during normal walking, this indicated equivalence.

Since the values of the projected conditions were compared with the values of the real condition within each participant, the parameters were not normalized by the participants’ anthropometric data.

### Ethics approval and consent to participate

According to the clarification of responsibility (Req-2018-00657) by the ethics committee of the Canton of Zurich, the project did not fall within the scope of the Human Research Act. As all participants were younger than 18 years of age, written informed consent was given by them and their parents.

## Results

### Participants

Twenty healthy children and adolescents (12 females, eight males) with a mean age of 10.9 ± 3.3 years (range 6-17y) participated. Care was taken to ensure that at least one participant could be recruited for each 1-year age stratum. Fifteen children were between 6 and 12 years old, and five were between 13 and 17. The mean height of the participants was 1.40 ± 0.18 m, and their mean weight was 34.9 ± 14.5 kg. Except for one participant, who wore glasses during the measurements, all participants had good visual acuity.

### Trials

To obtain eight valid trials for the two obstacles and the two conditions each, the participants performed between 41 and 80 trials.

### Tree trunk

#### Spatiotemporal parameters

When approaching the projected compared to the real tree trunk, there was a trend towards a slower but non-significant difference in gait velocity, as the 95% CI lay within a normal range of test–retest variability. Also, the step length was equivalent between the two conditions, although the TL was placed closer to the projected than the real tree trunk.

In the execution phase, when the tree trunk is crossed, there were no statistically significant differences in step or stride lengths, although there was a tendency towards smaller values for the projected condition. This tendency was confirmed by the foot placement after the tree trunk, which was closer to the projected than the real trunk. With the 95% CI exceeding the reference boundaries from literature, the step width was slightly broader when crossing the projected compared to the real tree trunk. The maximal toe clearance was significantly decreased in both limbs. The medians with their interquartile range (IQR) for the real and the projected conditions and the differences of the medians between the two conditions for all spatiotemporal parameters are presented in Table 2.

**Table 2:** Equivalence analyses and differences between the medians of the tree trunk scenario.

Phase	Parameters	Median [IQR] projected condition	Median [IQR] real condition	Difference between the medians (95% CI)	Reference boundaries from literature^37,38^
Preparation	Velocity (m/s)	1.57 [1.28; 1.69]	1.57 [1.46; 1.84]	**− 0.02 (− 0.12, 0.02)**	**± 0.14**
	Step length (m)	0.53 [0.50; 0.58]	0.52 [0.48; 0.58]	**0.00 (− 0.03, 0.00)**	**± 0.04**
Execution	Step length (m)	0.62 [0.57; 0.67]	0.62 [0.59; 0.67]	**− 0.02 (− 0.04, 0.01)**	**± 0.04**
	Step width (m)	− 0.01 [− 0.09; 0.05]	0.03 [− 0.09; 0.06]	− 0.01 (− 0.02, 0.00)	± 0.01
	Stride length (m)	1.15 [1.09; 1.26]	1.16 [1.07; 1.26]	**− 0.01 (− 0.05, 0.02)**	**± 0.07**
	Toe clearance LL (m)	0.08 [0.07; 0.10]	0.29 [0.28; 0.30]	− 0.20 (− 0.22, − 0.18)	*
	Toe clearance TL (m)	0.08 [0.07; 0.10]	0.27 [0.26; 0.28]	− 0.19 (− 0.21, − 0.17)	*
	JA ankle min LL (°)	− 17.79 [− 21.51; − 13.26]	− 27.79 [− 32.78; − 23.97]	8.96 (4.59, 11.83)	± 5.91
	JA ankle min TL (°)	− 23.31 [− 26.15; − 14.32]	− 21.76 [− 28.43; − 11.50]	**1.15 (− 3.34, 3.51)**	**± 5.91**
	JA ankle max LL (°)	15.60 [14.33; 17.31]	17.91 [14.14; 19.20]	**− 1.19 (− 1.69, − 0.52)**	**± 4.26**
	JA ankle max TL (°)	14.75 [12.06; 17.00]	15.06 [12.48; 17.74]	**− 0.09 (− 1.94, 1.62)**	**± 4.26**
	JA knee max LL (°)	71.26 [67.84; 76.49]	103.21 [97.82; 109.84]	− 34.29 (− 39.40, − 25.78)	± 6.46
	JA knee max TL (°)	67.84 [62.46; 74.25]	114.14 [106.86; 122.65]	− 45.47 (− 50.31, − 38.61)	± 6.46
	JA hip max LL (°)	46.95 [43.89; 48.64]	74.50 [71.55; 77.59]	− 27.64 (− 32.50, − 25.19)	± 6.82
	JA hip max TL (°)	41.85 [37.13; 44.51]	57.29 [51.07; 62.16]	− 14.79 (− 20.70, − 10.19)	± 6.82
Additional parameters	Distance pre tree (m)	0.13 [0.11; 0.16]	0.14 [0.13; 0.17]	− 0.02 (− 0.03, − 0.01)	–
	Distance post tree (m)	0.13 [0.09; 0.17]	0.21 [0.19; 0.22]	− 0.09 (− 0.10, − 0.05)	–

The differences between the medians were calculated by subtracting the real condition from the projected condition. Bold parameters are within the normal test–retest variability found in the literature for spatiotemporal parameters (children 6–7 years and young adults 21–35 years^37^) and kinematic parameters (children and adolescents 4–17 years^38^). Please note that the medians of the differences do not necessarily give the same results as when the medians of the projected condition are subtracted from the medians of the real condition.

*JA* joint angle, *LL* leading limb, *TL* trailing limb, *IQR* interquartile range, *CI* confidence interval.

*Dependent on obstacle height and directly linked to the maximal knee joint angle.

#### Kinematic parameters

**Figure 2:**
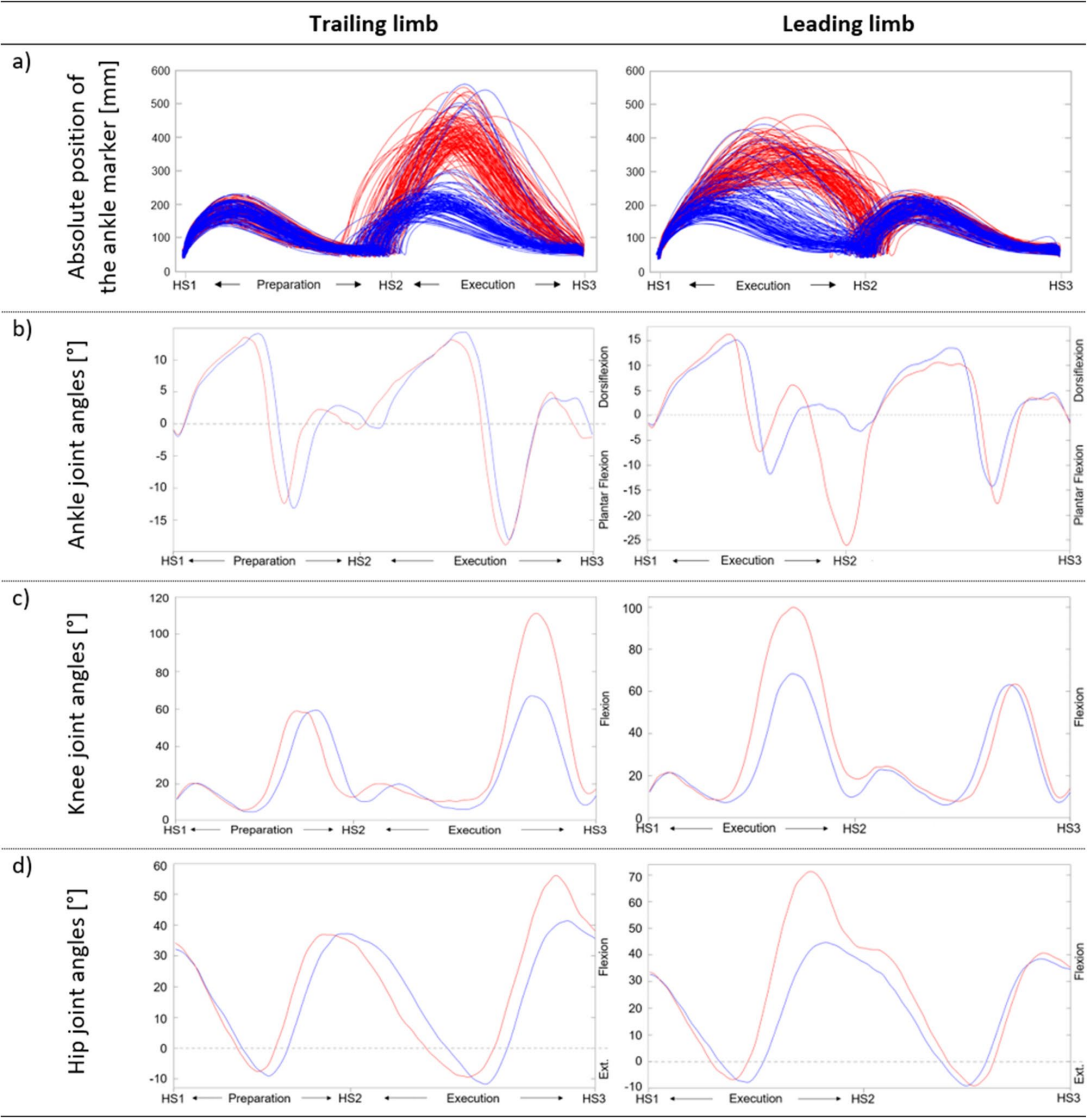
Trajectories of the ankle markers and joint angles of the tree trunk scenario. Comparison of the vertical trajectories of the ankle markers (**a**) and the median joint angles of the ankle, knee, and hip joints (**b**–**d**) of the leading and the trailing limb while mastering the tree trunk scenario for the projected (blue) and real (red) condition. *Abbreviations Ext.* extension, *HS* heel strike.

Due to technical and spatial constraints, only the TL could be measured in the preparation phase. Nevertheless, the overall patterns of the ankle marker trajectories were congruent for both conditions (Fig. 2a). Apart from a slight shift between the curves because of the different foot placement in the two conditions, also the joint angles were similar during this phase (Fig. 2b).

In the execution phase, the patterns of the ankle marker trajectories differed considerably between the two conditions. Only single trajectories in the projected condition reflected the observed pattern during the real condition and more often for the LL than for the TL (Fig. 2a). When performing the projected condition, the mean plantarflexion of the LL decreased by approximately 22° compared to the real condition at the end of the execution phase, when the foot was placed on the floor after the tree trunk. Although dorsiflexion of the LL decreased slightly (Fig. 2b) and was within a normal range of test–retest variability, the difference was still significant (Table 2). The ankle joint angles of the TL were similar for almost the whole gait cycle. The only difference was found towards the end of the execution phase when the foot remained in dorsiflexion in the projected condition compared to a plantarflexion position as measured in the real condition (Fig. 2b). Also, the knee and hip joint angles demonstrated that both feet were lifted less when stepping over the projected tree trunk. The difference of the maximal knee joint angle was thereby smaller for the LL than for the TL (Fig. 2c). The maximal hip joint angle, in contrast, differed less for the TL than for the LL (Fig. 2d). The medians with their IQR for the real and the projected conditions and the differences of the medians between the two conditions for all kinematic parameters are presented in Table 2.

### Kerbstones

#### Spatiotemporal parameters

The kerbstones scenario started directly with the execution phase, involving two gait cycles for both limbs. While gait velocity was measured over both gait cycles, all other spatiotemporal parameters were analysed for the first gait cycle only.

Step length, step width, and single support time were equivalent in both conditions. There was a slight difference in gait velocity while balancing on the kerbstones between the conditions. Still, it did not reach statistical significance and was within a normal test–retest variability. The medians with their IQR for the real and the projected conditions and the differences of the medians between the two conditions for all spatiotemporal parameters are presented in Table 3.

**Table 3:** Equivalence analyses and differences between the medians of the kerbstones scenario.

Gait cycle	Parameters	Median [IQR] projected condition	Median [IQR] real condition	Difference between the medians (95% CI)	Reference boundaries from literature^37,38^
Both gait cycles	Velocity (m/s)	0.48 [0.37; 0.57]	0.50 [0.36; 0.56]	**0.02 (− 0.08, 0.03)**	**± 0.14**
Gait cycle 1	Step length (m)	0.46 [0.42; 0.51]	0.44 [0.41; 0.48]	**0.01 (0.00, 0.02)**	**± 0.04**
	Step width (m)	− 0.01 [− 0.01; − 0.01]	− 0.01 [− 0.01; 0.00]	**0.00 (0.00, 0.00)**	**± 0.01**
	Single support time (s)	0.42 [0.36; 0.44]	0.39 [0.36; 0.42]	**0.01 (− 0.01, 0.02)**	**± 0.03**
	JA ankle min LL (°)	− 12.60 [− 15.98; − 10.67]	− 15.18 [− 20.85; − 9.35]	**1.13 (− 0.94, 3.67)**	**± 5.91**
	JA ankle min TL (°)	− 14.12 [− 19.42; − 11.27]	− 14.16 [− 17.05; − 11.65]	**0.33 (− 3.41, 1.75)**	**± 5.91**
	JA ankle max LL (°)	16.02 [12.59; 18.61]	16.10 [11.83; 18.27]	**0.08 (− 0.83, 1.16)**	**± 4.26**
	JA ankle max TL (°)	15.81 [13.37; 16.94]	14.35 [12.56; 15.37]	**0.52 (− 0.22, 2.33)**	**± 4.26**
Gait cycle 2	JA ankle min LL (°)	− 14.86 [− 19.50; − 10.18]	− 24.27 [− 27.88; − 19.57]	9.05 (4.30, 11.73)	± 5.91
	JA ankle min TL (°)	− 18.64 [− 22.58; − 12.10]	− 11.99 [− 15.03; − 9.53]	− 6.30 (− 8.07, − 4.08)	± 5.91
	JA ankle max LL (°)	15.63 [11.69; 17.47]	13.44 [11.21; 16.24]	**1.04 (− 0.29, 2.07)**	**± 4.26**
	JA ankle max TL (°)	14.05 [11.45 15.61]	19.26 [17.18; 22.89]	− 6.23 (− 9.52, − 4.89)	± 4.26

The differences between the medians were calculated by subtracting the real condition from the projected condition. Bold parameters are within a normal test–retest variability found in the literature for spatiotemporal parameters (children 6–7 years and young adults 21–35 years^37^) and kinematic parameters (children and adolescents 4–17 years^38^). Please note that the medians of the differences do not necessarily give the same results as when the medians of the projected condition are subtracted from the medians of the real condition.

*JA* joint angle, *LL* leading limb, *TL* trailing limb, *IQR* interquartile range, *CI* confidence interval.

#### Kinematic parameters

**Figure 3:**
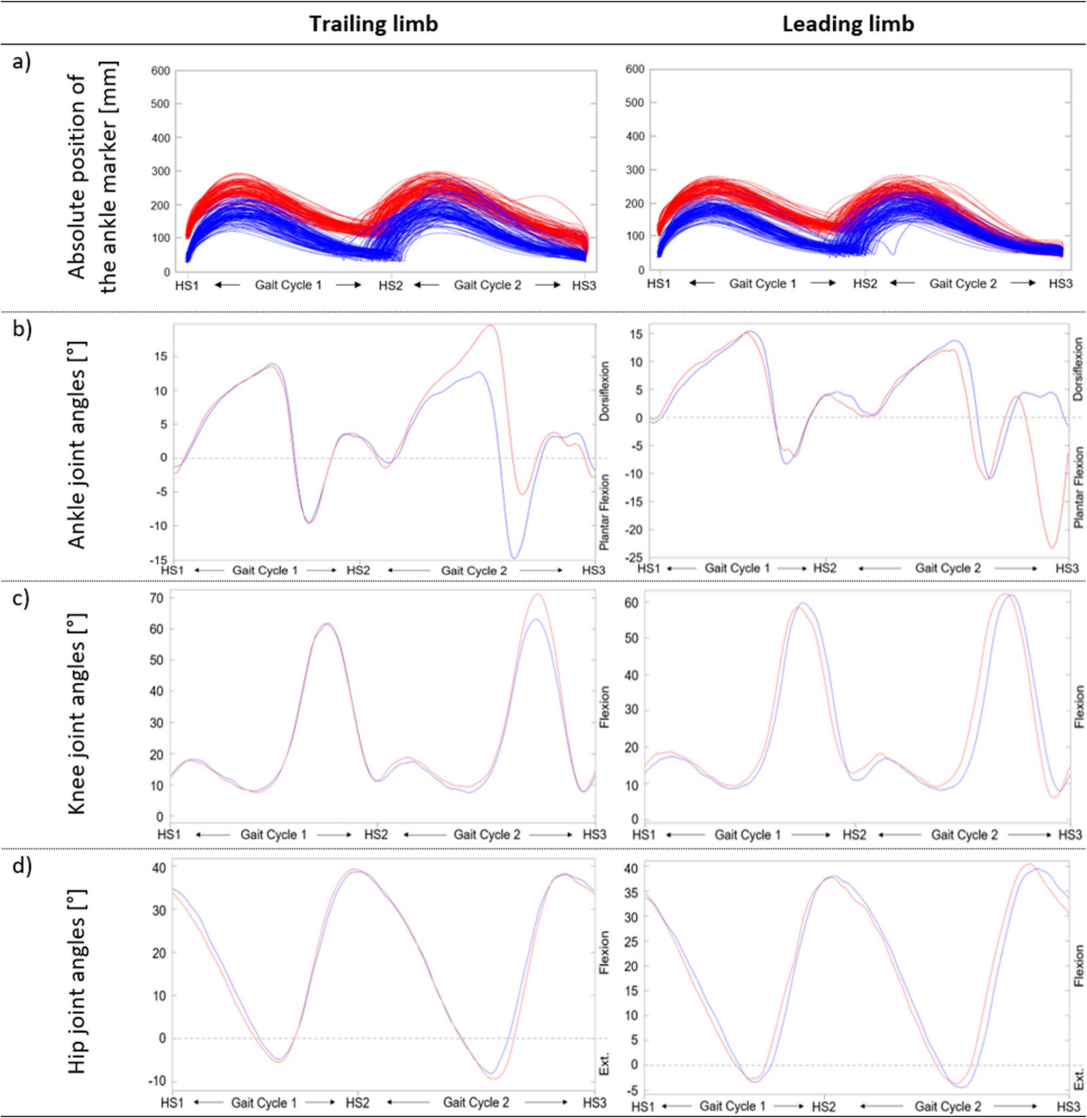
Trajectories of the ankle markers and joint angles of the kerbstones scenario. Comparison of the vertical trajectories of the ankle markers (**a**) and the median joint angles of the ankle, knee, and hip joints (**b**–**d**) of the leading and the trailing limb while mastering the kerbstones scenario in the projected (blue) and real (red) condition. *Abbreviations Ext.* extension, *HS* heel strike.

The overall pattern of the ankle marker trajectories was congruent over both conditions (Fig. 3a). The only difference was that the trajectories started at a higher position in the real condition due to the 0.07 m height of the real kerbstones, while the projected kerbstones were flat on the ground. In a few cases, the participants lifted the feet higher while balancing on the projected kerbstones, represented by those trajectories of the projected condition that reached a similar height as those of the real condition.

All joint angles of the LL and TL were approximately congruent in both conditions and during both gait cycles. Only towards the end of the second gait cycle, when the participants stepped down from the real kerbstones while they were already on the ground in the projected condition, there were some differences observable between the two conditions: While the foot of the LL was in a plantarflexion of about 23° in the real condition, it was dorsiflexed in about 5° in the projected condition (Fig. 3b). The TL showed a decreased dorsiflexion and an increased plantarflexion. The maximal knee joint angle was decreased by about 10° in the projected compared to the real condition due to the height difference of the kerbstones (Fig. 3b,c). There were no differences in the hip joint angle for the TL. However, for the LL there was first a small decrease of about 2° and then an increase of 3° when the participants stepped down from the projected compared to the real kerbstones (Fig. 3d). The medians with their IQR for the real and the projected conditions and the differences of the medians between the two conditions for all kinematic parameters are presented in Table 3.

### Equivalence Analyses of both Scenarios

According to the equivalence analysis, which assessed whether the spatiotemporal and kinematic parameters during task execution were similar in both conditions, no parameter was equivalent. However, when comparing the deviations between the conditions with existing evidence, the spatiotemporal parameters of both scenarios were within a normal range of test–retest variability. The kinematic parameters, in contrast, clearly exceeded the reference values, as expected, especially for the tree trunk scenario.

## Discussion

The aim of this project was to investigate whether healthy children and adolescents master real and projected obstacles in a similar way regarding various lower limb spatiotemporal and kinematic gait parameters. Using equivalence testing, we found 16 out of 25 parameters that were equivalent between the real and the projected conditions, as their CIs were within normal test–retest variability found in literature^37,38^.

As hypothesized, for both scenarios, the difference in gait velocity between the two conditions was within normal test–retest variability that has been reported for healthy children while walking at a self-selected speed on a simple walkway or a GAITRite® track^37,39^. The tendency towards a slower gait velocity in the tree trunk scenario supports the findings of Binaee and Diaz^24^. They found a slower approaching speed in a condition with projected obstacles, which might be explained by insecurity of the participants regarding how they should interpret the dimensions of the projected obstacle. However, for the kerbstones scenario, we found a contrary trend, which probably has been caused by the missing height dimension in the projected condition, and the participants might have felt more secure while balancing on the kerbstones.

Also, the differences in step length between the conditions remained within the normal test–retest variability for both scenarios^37,39,40^. The same appears true for the spatiotemporal parameters stride length, measured in the tree trunk scenario, and single support time in the kerbstones scenario. Both parameters revealed differences between the conditions that were within previously reported test–retest variability in healthy children^37^ and young women^40^. Apart from the step width, which was minimally broader in the real tree trunk condition, the differences of all these spatiotemporal parameters measured in our study were within previously reported boundaries. This indicates that the two projected obstacles indeed reflected the length and width dimensions of the real scenarios. Inversely, it also demonstrates that with projected obstacles, a gait pattern similar to mastering a real obstacle can be induced, at least when quantified by the parameters assessed in this study. However, for the height dimension, this is another story. Obviously, the height dimension of the tree trunk was absent in the floor projections and the three-dimensional appearance of the trunk was lost when the participants approached. As spatiotemporal parameters are adjusted while approaching an obstacle^41^, the 1.5 m between the point of highest congruence in appearance between the real and the projected obstacle in our study might have been enough for the participants to adapt their gait pattern. Consequently, toe clearance of both limbs was, as hypothesized, significantly decreased during the execution phase of the projected condition. The decrease in toe clearance was even greater than the height of the real tree trunk, which might indicate that the participants applied an additional safety margin when stepping over the real tree trunk. Chou and Draganich^42^ measured an obstacle height independent safety margin of 0.146 m for the TL while it was much smaller for unobstructed walking (0.031 m). A consistent minimum clearance distance between the lead foot and obstacles of different heights was reported by Binaee and Diaz^24^, who compared physical and AR obstacle crossing. They found a slightly greater toe clearance in the AR condition. As with gait speed, the authors explained this difference with the increased uncertainty of the participants regarding the AR obstacle height. The lower toe clearance in the projected condition is another indication that the projected tree trunk was not perceived as three-dimensional at the time of crossing. Therefore, the marker trajectories of the ankle joint markers corresponded more with the gait pattern of level walking than passing the real tree trunk.

In line with the results for the toe clearance, the maximal knee and hip joint angles were also, as hypothesized, significantly smaller in the projected compared to the real condition of the tree trunk scenario. The comparison with normal gait data of 4–17 years-old healthy children from two centres revealed that the decrease in knee and hip joint angles of both legs in our study exceeded the mean standard deviations during level walking (SD; 6.46° for maximal knee joint angle; 6.82° for maximal hip joint angle)^38^. The differences in the decrease of the joint angles in the LL and TL can be explained as follows: As the foot of the TL was placed closer to the tree trunk than the LL, the knee joint of the TL had to be flexed more than the knee of the LL when stepping over the real tree trunk. This strategy was not necessary for the projected trunk, as it could be safely passed without an increase in knee flexion, which, according to Chou and Draganich^42^, is the primary determinant for maintenance of constant toe clearance during obstacle crossing for the TL. Therefore, the difference in the knee joint angles between the two conditions was higher for the TL than for the LL. In contrast, the hip joint of the LL was flexed more than the TL to step over the real tree trunk. Therefore, the difference between the two conditions was greater for the LL than the TL. Our results show that toe clearance during obstacle crossing cannot be trained with such floor projections like we used in our study, as the movement pattern did not correctly represent the one which was observed in the real condition of our study. Consequently, this would also influence obstacle crossing in daily life. In practical terms, this would for example mean that kerbstone edges on streets or other small obstacles might not be passed safely, as it was never trained to lift the foot up to the toe clearance which is needed to cross the encountered obstacle. When the participants balanced over the real and the projected kerbstones, the knee and hip joint angles were visually comparable, as the children and adolescents used the same balancing strategy in both situations. Also this finding confirms our hypothesis.

Although we observed that only a few of the ankle trajectories of the projected tree trunk condition corresponded with those of the real condition, the picture was different for the two limbs. The trajectories of the LL were more often comparable between the two conditions than those of the TL when passing the tree trunk. This result coincides with Huang et al., who found a lower success rate for the TL than for the LL when crossing a virtual obstacle^25^. Other studies including real and virtual obstacles supported this finding and argued with the theory of a vision-dependent LL and memory-dependent TL^43–45^. This means that the LL is visible in the lower visual field while crossing the obstacle. The TL, in contrast, is outside the field of view. Thus, the participants must rely on the previously memorized obstacle characteristics (e.g., height, length, width) to successfully complete the task^44^.

The differences in ankle joint angles between the two conditions in the execution phase of both scenarios exceeded the calculated minimal (plantarflexion 5.91°) and maximal (dorsiflexion 4.26°) mean SD during level walking^38^. Due to different foot placement strategies, the projected condition in the tree trunk scenario yielded a huge decrease in plantarflexion of the leading foot after obstacle crossing. While the participants first touched the ground after the real tree trunk with their forefoot, first contact happened with the heel in the projected condition instead. The participants chose a similar strategy when stepping down from the real kerbstones. Consequently, the projected kerbstones resulted in dorsiflexion instead of plantarflexion for the LL and a decrease in dorsiflexion for the TL. Thus, our hypothesis of similarity for minimal and maximal ankle joint angles in the kerbstone scenario was not confirmed for the second gait cycle. However, in the first gait cycle, where the participants balanced on the kerbstones on one level, the parameters were within a normal range of test–retest variability, and balancing was not affected by the height difference in the two conditions.

### Limitations

We included 20 youths and deliberately selected participants with a broad age range to represent the age range of the patients in our rehab centre. Therefore, we cannot generalize our results. However, our findings indicate that the statistical design, including an equivalence analysis with a within-subject design, and the sample size were appropriate. Our results revealed that most of the assessed parameters are equivalent between the projected and the real condition. The fact that this is true for our limited and heterogeneous population makes the results all the more compelling. It demonstrates that the results are robust, and parameters are stable for the real and the projected scenarios. Almost all the confidence intervals of the parameters lie either within the boundaries (equivalence margin) or clearly outside. In fact, for the latter, it can be observed that the parameters’ point estimates are well outside the boundaries. Therefore, the findings of the study would still be the same, despite the confidence intervals becoming smaller with a more homogenous group or a larger study population. We observed during the measurements that younger participants tended to need more repetitions to reach eight valid trials than older participants. This high repetition rate was exhausting for the children and may also have influenced the trial execution. However, as two-thirds of the parameters had their CIs within the reference boundaries and the CIs of the other parameters clearly exceeded those boundaries, we were able to show that the differences between the conditions were based on technical constraints and not on the fatigue of the participants. Furthermore, even though the variability of gait parameters is higher in younger children due to the still ongoing of gait control^46^, we could not find a trend of age on the tested parameters. We could not find any reference values for obstacle crossing tasks in our study population’s age range. Therefore, our reference boundaries derive from studies that evaluated level ground walking in healthy children, adolescents, and young women. However, as we expected a higher variability during obstacle crossing compared to level ground walking, we considered the used reference boundaries as an even more conservative approach.

The technical and methodological setup displayed additional limitations. The dimensions of the obstacles were not adapted to the participants’ anthropometric characteristics. However, as this was the case for the projected and the real obstacles, it should not have influenced the results. With the equipment available, a continuous adaptation of the obstacles’ appearance while the participants approached was not possible. Thus, we had to find a compromise for the optimal starting point in the tree trunk scenario. The projected tree trunk should have a three-dimensional appearance, while the distance had to be long enough to allow the measurement of at least one gait cycle for the preparation phase. However, the measurement of only one gait cycle precludes drawing any conclusions on possible gait adaptations that might have occurred further away from the obstacle. Given the technological advances, a future study could be performed with AR or VR glasses, which would enable a three-dimensional representation of the scenarios. Furthermore, AR and VR could open the door to new therapeutic approaches, for example, by enabling training of complex everyday life walking activities in a secure and easily accessible environment. Finally, the presented results refer to healthy children and adolescents only. Given that the aim is to apply floor projections in the clinical setting, it is essential to investigate their effect on gait parameters in the patient populations of interest.

## Conclusion

In our study, we found some similarities between conditions in spatiotemporal and kinematic gait parameters. However, it was evident that obstacles with a vertical extension were not applicable for floor projections generated by a standard projector only, as the three-dimensional effect of the obstacles got lost when the participants approached them. Consequently, it was not possible to correctly reflect the height dimension of the real obstacle in the projected condition. Differences in kinematics when mastering real and projected obstacles, therefore, exceeded reported test–retest variability as soon as specific adaptations were required to successfully step over (tree trunk) or down from (kerbstones) the obstacles. Exercises aiming to improve toe clearance or knee and hip flexion should, thus, favourably be trained with real obstacles. A further promising approach might be more advanced projections, as used, for example, in the study of Binaee and Diaz, who used stereoscopic goggles and motion-tracking to adapt the projection’s perspective to the subjects’ head position^24^. AR or VR glasses could be another promising approach to present the obstacles in a more realistic way. It was no problem, however, to represent the correct width and length dimensions of the real obstacle in the projected condition. Therefore, the differences between the two conditions were in a normal range of test–retest variability for the spatiotemporal parameters like gait velocity, step and stride length, and step width (only kerbstones). Consequently, our results indicate that the use of floor projections would be most suitable to train a precise foot placement, as in our scenario with the kerbstones, or to work on the step length, like in our tree trunk scenario. However, to implement floor projections as therapy approach in the clinical setting, a similar study should be performed with a patient population to reveal whether the results would be the same. A combination of floor projections and real obstacles, thus creating an everyday-like environment, might also be an interesting option to train gait activities.

## Data Availability

The dataset collected and analysed during the current study is available from the corresponding author on request.

## References

[1] Katz-Leurer M, Rotem H, Keren O, Meyer S (2009). Balance abilities and gait characteristics in post-traumatic brain injury, cerebral palsy and typically developed children. Dev. Neurorehabil..

[2] Gandhi P, Chan K, Verrier MC, Pakosh M, Mussleman KE (2017). Training to improve walking after pediatric spinal cord injury: A systematic review of parameters and walking outcomes. J. Neurotrauma.

[3] Karalok ZS (2018). Risk factors and motor outcome of paediatric stroke patients. Brain Dev..

[4] Vargus-Adams JN, Martin LK (2011). Domains of importance for parents, medical professionals and youth with cerebral palsy considering treatment outcomes. Child Care Health Dev..

[5] van Hedel HJA, Meyer-Heim A, Rüsch-Bohtz C (2016). Robot-assisted gait training might be beneficial for more severely affected children with cerebral palsy. Dev. Neurorehabil..

[6] Beretta E, Romei M, Molteni E, Avantaggio P, Strazzer S (2015). Combined robotic-aided gait training and physical therapy improve functional abilities and hip kinematics during gait in children and adolescents with acquired brain injury. Brain Inj..

[7] Oh, Y. & Yang, S. Defining exergames & exergaming. In *21–23 October 2010 Meaningful Play*, Michigan, USA (2015).

[8] Schuler T, Brütsch K, Müller R, van Hedel HJ, Meyer-Heim A (2011). Virtual realities as motivational tools for robotic assisted gait training in children: A surface electromyography study. NeuroRehabilitation.

[9] Ricklin S, Meyer-Heim A, van Hedel HJA (2018). Dual-task training of children with neuromotor disorders during robot-assisted gait therapy: Prerequisites of patients and influence on leg muscle activity. J. Neuroeng. Rahabil..

[10] van Hedel HJA, Aurich T, Reinkensmeyer DJ, Dietz V (2016). Clinical application of rehabilitation technologies in children undergoing neurorehabilitation. Neurorehabilitation Technology.

[11] Keller U, van Hedel HJA, Klamroth-Marganska V, Riener R (2016). ChARMin: The first actuated exoskeleton robot for pediatric arm rehabilitation. IEEE ASME Trans. Mechatron..

[12] Vidrios-Serrano, C., Bonilla, I., Vigueras-Gómez, F. & Mendoza, M. Development of a haptic interface for motor rehabilitation therapy using augmented reality. In *25–29 August 2015 37th Annual International Conference of the IEEE Engineering in Medicine and Biology Society (EMBC)*, Milan, Italy 1156–1159. 10.1109/EMBC.2015.7318571 (2015). 10.1109/EMBC.2015.7318571 26736471

[13] de Assis GA, Corrêa AG, Rodrigues Martins MB, Pedrozo WG, de Deus Lopes R (2016). An augmented reality system for upper-limb post-stroke motor rehabilitation: A feasibility study. Disabil. Rehabil. Assist. Technol..

[14] Cipresso P, Giglioli IAC, Raya MA, Riva G (2018). The past, present, and future of virtual and augmented reality research: A network and cluster analysis of the literature. Front. Psychol..

[15] Glegg S (2017). Virtual rehabilitation with children: Challenges for clinical adoption [from the field]. IEEE Pulse.

[16] Olivieri I (2018). Computer Assisted REhabilitation (CARE) Lab: A novel approach towards pediatric rehabilitation 2.0. J. Pediatr. Rehabil. Med..

[17] Bennour S, Ulrich B, Legrand T, Jolles BM, Favre J (2018). A gait retraining system using augmented-reality to modify footprint parameters: Effects on lower-limb sagittal-plane kinematics. J. Biomech..

[18] Rossano C, Terrier P (2016). Visually-guided gait training in paretic patients during the first rehabilitation phase: Study protocol for a randomized controlled trial. Trials.

[19] Espay AJ (2010). At-home training with closed-loop augmented-reality cueing device for improving gait in patients with Parkinson disease. J. Rehabil. Res. Dev..

[20] Lee J, Yoo HN, Lee BH (2017). Effects of augmented reality-based Otago exercise on balance, gait, and physical factors in elderly women to prevent falls: A randomized controlled trial. J. Phys. Ther. Sci..

[21] Yoo HN, Chung E, Lee BH (2013). The effects of augmented reality-based Otago Exercise on balance, gait, and falls efficacy of eldery women. J. Phys. Ther. Sci..

[22] World Health Organization (2007). International Classification of Functioning, Disability and Health for Children and Youth: ICF-CY. 45.

[23] Rosenbaum P, Gorter JW (2012). The 'F-words' in childhood disability: I swear this is how we shoud think. Child Care Health Dev..

[24] Binaee K, Diaz GJ (2019). Assessment of an augmented reality apparatus for the study of visually guided walking and obstacle crossing. Behav. Res. Methods.

[25] Huang CK (2019). An altered spatiotemporal gait adjustment during a virtual obstacle crossing task in patients with diabetic peripheral neuropathy. J. Diabetes Complic..

[26] Argelaguet Sanz, F., Olivier, A. H., Bruder, G., Pattré, J. & Lécuyer, A. Virtual proxemics: Locomotion in the presence of obstacles in large immersive projekction environments. In *2015 IEEE Virtual Reality (VR)*, Arles, France 75–80. 10.1109/VR.2015.7223327 (2015).

[27] Fink PW, Foo PS, Warren WH (2007). Obstacle avoidance during walking in real and virtual environments. ACM Trans. Appl. Percept..

[28] Palmisano C (2022). A fully-immersive virtual reality setup to study gait modulation. Front. Hum. Neurosci..

[29] jithurajeevt. Planeten Himmelskörper Wasser Baum Hintergrund. *pngtree*. Retreived 24.09.2018 (not accessible anymore). https://de.pngtree.com/freebackground/cartoon-h5-green-background_264998.html (n.d.).

[30] Yakovliev. Loch in der Mauer. Comic-Stil. *iStock by Getty Images*. Retrieved 24.09.2018. https://www.istockphoto.com/ch/vektor/loch-im-der-mauer-comics-stil-von-hand-gezeichnete-vektor-illustration-gm540101482-96364925?irgwc=1&esource=AFF_IS_IR_SP_ClipArtLogo.com_340407&asid=ClipArtLogo.com&cid=IS (2016).

[31] Kadaba MP, Ramakrishnan HK, Wootten ME (1990). Measurement of lower extremity kinematics during level walking. J. Orthop. Res..

[32] Stevens PM (2010). Clinimetric properties of timed walking events among patient populations commonly encountered in orthotic and prosthetic rehabilitation. J. Prosthet. Orthot..

[33] Deconinck FJA, Savelsbergh GJP, De Clercq D, Lenoir M (2010). Balance problems during obstacle crossing in children with Developmental Coordination Disorder. Gait Posture.

[34] McFadyen BJ, Malouin F, Dumas F (2001). Anticipatory locomotor control for obstacle avoidance in mid-childhood aged children. Gait Posture.

[35] Canty, A. & Ripley, B. *Boot: Bootstrap functions* (originally by Angelo Canty for S) (2017).

[36] Walker E, Nowacki AS (2011). Understanding equivalence and noninferiority testing. J. Gen. Intern. Med..

[37] Stolze H, Kuhtz-Buschbeck JP, Mondwurf C, Jöhn K, Friege L (1998). Retest reliability of spatiotemporal gait parameters in children and adults. Gait Posture.

[38] Pinzone O, Schwartz MH, Thomason P, Baker R (2014). The comparison of normative reference data from different gait analysis services. Gait Posture.

[39] Thevenon A (2015). Collection of normative data for spatial and temporal gait parameters in a sample of French children aged between 6 and 12. Ann. Phys. Rehabil. Med..

[40] Fryzowicz A, Murawa M, Kabacinski J, Rzepnicka A, Dworak LB (2018). Reference values of spatiotemporal parameters, joint angles, ground reaction forces, and plantar pressure distribution during normal gait in young women. Acta Bioeng. Biomech..

[41] Chou LS, Draganich LF (1998). Placing the trailing foot closer to an obstacle reduces flexion of the hip, knee, and ankle to increase the risk of tripping. J. Biomech..

[42] Chou LS, Draganich LF (1997). Stepping over an obstacle increases the motions and moments of the joints of the trailing limb in young adults. J. Biomach..

[43] Heijnen MJ, Muir BC, Rietdyk S (2012). Factors leading to obstacle contact during adaptive locomotion. Exp. Brain. Res..

[44] Heijnen MJ, Romine NL, Stumpf DM, Rietdyk S (2014). Memory-guided obstacle crossing: More failures were observed for the trail limb versus lead limb. Exp. Brain. Res..

[45] Lajoie K, Bloomfield LW, Nelson FJ, Suh JJ, Marigold DS (2012). The contribution of vision, proprioception, and efference copy in storing a neural representation for guiding trail leg trajectory over an obstacle. J. Neurophysiol..

[46] Gouelle A, Leroux J, Bredin J, Mégot F (2016). Changes in gait variability from first steps to adulthood: Normative data for the gait variability index. J. Mot. Behav..

